# Editorial: Bioactive bone regenerative materials and bionic prosthesis interfaces

**DOI:** 10.3389/fbioe.2022.1111743

**Published:** 2022-12-13

**Authors:** He Liu, Yuhang Tian, Chao Zhao, Jianxun Ding

**Affiliations:** ^1^ Department of Orthopedics, The Second Hospital of Jilin University, Changchun, China; ^2^ Department of Chemical and Biological Engineering, The University of Alabama, Tuscaloosa, AL, United States; ^3^ Key Laboratory of Polymer Ecomaterials, Changchun Institute of Applied Chemistry, Chinese Academy of Sciences, Changchun, China

**Keywords:** bioactive materials, bone regenerative materials, bionic prosthesis interfaces, controlled drug delivery, regenerative medicine

Bone defects caused by high-energy trauma, infection, and tumor reconstruction have been a severe clinical challenge and placed a significant burden on patients. Although autografting has been recognized as the gold standard for the treatment of bone defects, it still has many unavoidable disadvantages. The development of bioactive bone regeneration materials has brought innovations to the reconstruction of skeletal functions.

The bone regeneration biomaterials should have the appropriate biocompatibility and enhanced mechanical, antibacterial, and osteogenic properties. When bioactive bone regeneration materials are implanted into the defect site, they should promote integration of the prosthetic interface with the surrounding bone tissue and maintain long-term stability, which is essential for bone repair.

Herein, this Research Topic covered 21 publications submitted by 151 researchers, comprising 11 original research articles and 10 review articles (total views: 26196 views as of 10 December 2022). These studies included antibacterial materials for dental implant surgery, bone tissue engineering, drug release system, and finite element analysis. The papers included in this topic are briefly discussed below.

In dental implant surgery, ideal osteointegration of the implant with the surrounding bone tissue provides the basis for bone regeneration (Wang et al.) However, the biofilm formed on the prosthesis interface after infection destroys the bone integration ability and severely affects the progress of bone repair. Therefore, it is necessary to develop antibacterial biomaterials to prevent and treat peri-implantitis. α-Amylase (α-Amy) effectively destroyed the biofilm of *Actinomyces viscosus* by decomposing the complex polysaccharide. However, 0.1%–0.5% of α-Amy showed significant toxicity to MC3T3-E1 cells. D-arginine was investigated to enhance the biofilm-decomposing ability of 0.01% α-Amy without any cytotoxicity by augmenting the catalytic triad and stabilizing the calcium binding region (Li et al.) Moreover, biomaterials with remineralization property are also essential in the dental implant surgery, especially in dental caries. These advanced materials, including inorganic, organic, and polymer materials, have the ability of reconstructing re-mineralized tissue on damaged surfaces while enhancing mechanical strength at the same time. The detailed classification and functions of these materials were reviewed (Xu et al.) Apart from the single anti-infection or osteogenic property, the dual functional modification of dental implant surface with both antibacterial and osteogenic properties, such as bio-macromolecule, polymer, titanium dioxide (TiO_2_) nanotube, and metal ion/nanoparticle coatings, and their effect on soft tissue integration, bone regeneration, and immune response were also summarized (Wang et al.). In addition to biomaterials, the laser-assisted implant for bone repair has a good development prospect. Er:YAG laser is a kind of dental hard tissue laser approved by the American Food and Drug Administration (FDA). Ti alloy scaffold improved surface wettability, accelerate osteoblast adhesion, and generate new bone after Er:YAG laser therapy. Combining Er:YAG laser with Nd:YAG, another low-intensity laser promoting the osteoblast differentiation at early edge, achieved the best results in bone regeneration and implant tissue construction (Zhao and Li).

Bone tissue engineering integrates the traditional methods and ideas from materials engineering and biological science to produce the desired bone substitution. The scaffolds, cells, and regulatory factors are essential elements in bone tissue engineering. The tetraethyl orthosilicate-based supramolecular hydrogel was used as an ideal osteo-inductive factor to promote the osseointegration at the bone defect site and combined with poly(vinyl alcohol) and sodium tetraborate to accelerate osteogenic performance *in vitro* and *in vivo* (Zheng et al.) In the process of bone regeneration, neovascularization is also an essential element. Blood vessels served as a communication network between the new bone and surrounding tissues, maintaining the stability of new bone. The strontium-substituted calcium silicate (SrCS) has significant osteogenic and angiogenesis properties. The SrCS and silk fibroin composite scaffold was prepared, and its osteogenesis and angiogenesis were well evaluated for calvarial defect reconstruction (Zhou et al.) Besides, the platelet derivatives, including platelet-rich plasma (PRP), platelet-rich fibrin (PRF), and platelet lysates (PL), which are rich in growth factors and adhesion proteins were combined with biomaterial scaffolds for application in cartilage tissue engineering to address the limited self-renewal capacity of articular cartilage due to the lack of blood vessels and nerves (Wu et al.) Typically, natural biopolymers, such as collagen, silk, hyaluronic acid, chitosan, and extracellular matrix, for the repair of meniscal injury have also been well-reviewed (Peng et al.)

Interbody fusion surgery is a promising approach to the treatment of disc protrusion and spondylolisthesis. The three-dimensional (3D) printed biomaterial scaffolds realized the customized interbody fusion cage to achieve biological stability (Zhang et al.) In general, 3D printed implants, especially 3D printed Ti alloy, have been widely applied in orthopedics. However, the Ti alloy implant surfaces always need to be modified to achieve ideal osseointegration. Common modification methods include chemical surface modification, physical surface modification, and biological surface modification through which the prosthetic interface obtained the antibacterial property, enhanced osseointegration, and improved mechanical properties (Sheng et al.)

The sustained-release system for delivery of bioactive agents through a variety of biomaterials is an indispensable method for promoting bone regeneration. Mesoporous bioactive glass (MBG) was known as an excellent drug carrier due to its porosity and osteogenic property. Herein, the alginate-modified MBG was prepared to improve the loading efficiency and prolong the sustained release period (Yao et al.) Metal magnesium ion (Mg^2+^) promoted bone regeneration by regulating the behaviors of bone marrow mesenchymal stem cells. The magnesium-contained calcium phosphate cement was prepared for the sustained release of Mg^2+^ to achieve long-term mechanical stability and osteogenic property (Wu et al.) Moreover, co-delivery could be preferred for tumor therapy, and the intracellular co-delivery of proteins and small molecular antitumor drugs from nanocarriers was presented (Cheng et al.)

Chitosan, an emerging biomaterial, has been extensively used in bone regeneration due to its outstanding properties. The characteristics of chitosan and its derivatives, as well as the specific application forms in infected bone defects, were specifically summarized (Tian et al.) Yu et al. investigated the chitosan/hydroxyapatite/polycaprolactone composite scaffold to inoculate endothelial progenitor cells and bone marrow mesenchymal stem cells to evaluate the bone repair ability by detecting osteogenesis-related genes (Yu et al.) The results demonstrated that this dual-cell treatment was better than single-cell or cell-free treatments. Moreover, the application of exosomes to cure bone defects has some advantages compared to cell therapy, such as a wide range of sources and not limited to stem cells. The mechanisms of exosomes regulating osteogenesis, angiogenesis, and inflammation modulation were summarized (Zhang et al.)

In addition to the ability to integrate the prosthesis interface with the surrounding bone tissue, the biomechanical properties of designed prosthesis were important factors in promoting bone regeneration and functional reconstruction. In this Research Topic, several studies used finite element analysis to analyze the biomechanical distribution of 3D printed prostheses. After finite element verification, the different fixation methods of 3D printed prosthesis for the repair of metaphyseal bone defects resulted in different mechanical behaviors (Liu et al.) A unique bone mineral density screw was designed and compared with the traditional screw by finite element analysis method for mechanical properties and then improved by topology optimization (Zhou et al.) Furthermore, based on topology optimization, an optimized prosthesis with enhanced biomechanical properties was designed to repair the bone defect of proximal femur (Xue et al.) Vitallium Prosthesis, a customized 3D printed prosthesis was also designed and applied to the patients with ischemic necrosis of the talus and gained the expected results (Luo et al.)

Finally, the research hotspots of bone regeneration materials at each stage in the past 20 years, the latest progress of existing research, and the exploration direction for the future are comprehensively evaluated (Zhang et al.)

In summary, this invited Research Topic covered several fundamental and clinical studies with various bone regeneration biomaterials and bionic prosthesis interfaces. This interdisciplinary research has created an excellent theoretical and practical basis for the innovation of biomaterials and the transformation of clinical medicine, and also helps readers to better understand this field.

